# There is no dose–response relationship between the amount of exercise and improvement in HbA1c in interventions over 12 weeks in patients with type 2 diabetes: a meta-analysis and meta-regression

**DOI:** 10.1007/s00592-022-01918-8

**Published:** 2022-08-05

**Authors:** Elizabeth Wrench, Kate Rattley, Joel E. Lambert, Rebecca Killick, Lawrence D. Hayes, Robert M. Lauder, Christopher J. Gaffney

**Affiliations:** 1 Lancaster Medical School, Health Innovation One, Lancaster University, Sir John Fisher Drive, Lancaster, LA1 4AT UK; 2 East Lancashire Teaching Hospitals NHS Trust, Blackburn, BB2 3HH UK; 3 Institute of Clinical Exercise and Health Science, School of Health and Life Sciences, University of West of Scotland, Glasgow, G72 0LH UK; 4 Mathematics and Statistics, Lancaster University, Lancaster, LA1 4YG UK

**Keywords:** Type 2 diabetes, Aerobic exercise, Glycaemic control, HbA1c

## Abstract

**Aims:**

Aerobic exercise is well recognised as an effective treatment for people with type 2 diabetes but the optimal amount of aerobic exercise to improve glycaemic control remains to be determined. Thus, the aim of this meta-analysis and meta-regression was to assess the impact of volume and intensity of aerobic exercise on glycaemic control.

**Methods:**

Medline, Cochrane, Embase, and Web of Science databases were searched up until 15 December 2020 for the terms “aerobic exercise AND glycaemic control”, “type 2 diabetes AND exercise”, and “exercise AND glycaemic control AND Type 2 diabetes AND randomised control trial”. We included (i) randomised control trials of ≥ 12 weeks, (ii) trials where participants had type 2 diabetes and were aged 18 or over, and (iii) the trial reported HbA1c concentrations pre- and post-intervention. Two reviewers selected studies and extracted data. Data are reported as standardised mean difference (SMD) and publication bias was assessed using funnel plots.

**Results:**

A total of 5364 original titles were identified. Sixteen studies were included in the meta-analysis. Aerobic exercise reduced HbA1c versus control (SMD = 0.56 (95% CI 0.3–0.82), *p* < 0.001). There were also significant reductions in BMI (SMD = 0.76 (95% CI 0.25–1.27), *p* < 0.05). There was no dose–response relationship between improvement in HbA1c and the intensity and volume of the intervention (*p* > 0.05).

**Conclusions:**

Twelve-week or longer aerobic exercise programmes improve glycaemic control and BMI in adults with type 2 diabetes. Longer or more intense interventions appear to confer no additional benefit on HbA1c.

**Supplementary Information:**

The online version contains supplementary material available at 10.1007/s00592-022-01918-8.

## Introduction

### Rationale

Diabetes is a burden on global health systems with over 422 million cases worldwide, of which around 90% are type 2 [1–3]. It is predicted that due to the ageing population by 2038 there will be 20% more cases than in 2000, and this increase is expected to augment the economic burden by ~ 45% [4]. In the UK, diabetes will pose a major clinical and financial challenge to the National Health Service (NHS) with the annual cost of health care for diabetes treatment predicted to rise to £2.2 billion per annum by 2040, and this will contribute to around 17% of the NHS annual budget [4, 5].

In healthy individuals, beta cells are stimulated to release insulin to mediate uptake of glucose, amino acids, and fatty acids into tissue [6]. In type 2 diabetes, the development of insulin resistance ultimately promotes a reduction in insulin production and the failure of pancreatic beta cells [7]. A large majority of people suffering from type 2 diabetes are obese, with central visceral adiposity [8] and there is an inverse linear relationship between body mass index (BMI) and the age at which type 2 diabetes is diagnosed [9]. Beyond the age of 30, the human pancreas is unable to keep renewing beta cells and therefore loss of beta cells, such as due to glucolipotoxicity, explains why there is an increased risk of type 2 diabetes with age [6]. Whilst people with type 2 diabetes are treated pharmacologically with metformin, sulfonylureas and ultimately, insulin, nutritional [10] and aerobic exercise interventions [11] have proven highly effective at reducing hyperglycaemia. Aerobic exercise interventions are uniquely efficacious as they promote glucose uptake through a non-insulin-dependent mechanism that is intact in people with type 2 diabetes [11].

When at rest, the body requires insulin-dependent mechanisms to control glucose homeostasis [12]. Insulin signalling requires the activation of phosphatidylinositol 3-kinase (PI3K), phosphorylation of the insulin receptor, and the insulin receptor substrate-1/2 [13]. In type 2 diabetes, insulin resistance and the failure of PI3K-mediated glucose transporter type 4 (GLUT-4) translocation result in chronic hyperglycaemia [14]. Aerobic exercise does not require PI3K to prompt GLUT-4 translocation and thus, insulin resistant tissue will still take up glucose during exercise [15]. There have been a number of signalling pathways implicated in this process, including adenosine monophosphate-activated protein kinase (AMPK), calcium (Ca^2+^) /calmodulin-dependent protein kinases, and protein kinase C [13] that are reviewed elsewhere [16]. AMPK activity has been proposed as the central instigator of exercise-mediated glucose uptake [13] and thus, the most effective exercise regimens seem to be the ones that promote greatest increase in AMPK [17].

Chronic aerobic exercise training has been proven to improve body composition by increasing energy expenditure and reducing visceral fat [18]. Regular aerobic training increases both the expression and activity of proteins involved in insulin signalling pathways, therefore, improving tissue insulin sensitivity for up to 72 h post-exercise [11]. The elevated plasma glucose and lipid levels in type 2 diabetes can also be lowered through oxidation in regular aerobic exercise [19]. This helps to alleviate the glucotoxicity and lipotoxicity experienced by pancreatic beta cells, reducing inflammation and oxidative stress on tissues [19]. This allows damaged beta cells to recover and protects functional islets, preventing the worsening of endocrine function [19].

There are three key steps which allow aerobic exercise to increase glucose uptake by up to 50%, these are delivery, transport across the membrane, and oxidation causing intracellular influx [15]. Delivery of glucose to active muscle cells is increased due to a rise in blood flow and this is associated with an increase in capillary recruitment which subsequently increases the surface area for influx of glucose and oxygen [15]. Oxygen consumption is coupled to blood flow and this ensures sufficient glucose reaches active muscle tissue [15]. All of these factors induced during aerobic exercise allow glucose to be up taken into muscle cells, reducing the concentration of plasma glucose and therefore helping to reduce hyperglycaemia [12]. This therefore means aerobic exercise can be used as a non-pharmacological method to regulate glucose homeostasis in those with type 2 diabetes.

The optimal amount of aerobic exercise to improve glycaemic control in adults with type 2 diabetes remains incompletely understood. This meta-analysis and meta-regression investigated the association between volume and intensity of aerobic exercise on glycaemic control in adults with type 2 diabetes. The focus is specifically on aerobic exercise as aerobic and resistance exercise both utilise different mechanisms to improve glycaemic control, and there are also variations in the definitions of amount of exercise between the two. Resistance exercise increases muscle mass which increases GLUT-4 abundance without altering the muscle’s intrinsic pathways [11], and therefore the two mechanisms are somewhat distinct. Resistance exercise also typically uses progressive overload [20] so this means the intensity and volume would change over the duration of the intervention, making the amount of exercise difficult to compare between studies.

### Objectives

The benefits of aerobic exercise for type 2 diabetes are well documented, however, the volume and intensity of aerobic exercise required to improve glycaemic control are less well quantified. The aim of this systematic review and meta-analysis was to determine the impact of volume and intensity of aerobic exercise on glycaemic control in ≥ 12-week training studies in people with type 2 diabetes. Therefore, the effect of aerobic exercise training on the following outcomes was analysed—HbA1c, fasting blood glucose, BMI, triglycerides, cholesterol (total, high-density lipoprotein (HDL), and low-density lipoprotein cholesterol (LDL)), and the effect of intensity/volume of aerobic exercise training on HbA1c.

## Methods

### Eligibility criteria

This systematic review and meta-analysis were conducted in accordance with Preferred Reporting Items for Systematic Reviews and Meta-Analyses (PRISMA) guidelines [21]. EW and KR independently conducted the eligibility assessment in an unblinded and standardised manner. Where there was disagreement, CG acted as the final adjudicator. Once each database search was completed and manuscripts were sourced, all studies were downloaded into a single reference list with duplicates removed. Titles and abstracts were then screened for eligibility and full texts were only retrieved for studies with HbA1c and a supervised exercise intervention of ≥ 12 weeks. All studies retrieved as full texts were then assessed using the complete eligibility criteria with first and second authors confirming inclusion and exclusion. For four of these studies [22–25], the authors were contacted for supplemental data, of which zero replied. These four studies were therefore subsequently excluded [22–25].

### Information sources

A systematic literature search was conducted using the following databases: MEDLINE (accessed by PubMed), Cochrane Central Register of Controlled Trials, Embase, and Web of Science. The literature search was completed until 15 December 2020. The search was performed within all fields and terms were “exercise AND glycaemic control”, “type 2 Diabetes AND exercise”, and “exercise AND glycaemic control AND “type 2 diabetes AND randomised control trial”.

### Study selection

Studies that met the following criteria were included in this meta-analysis: (1) published as a full-text manuscript; (2) not a review; (3) participants had type 2 diabetes (age ≥ 18 years); (4) studies were required to employ an intervention design and include an aerobic exercise training period of ≥ 12 weeks. Additionally, descriptive data (e.g. sample size, mean, and standard deviation) reporting was required. Where this was not possible, details were requested from the authors. All studies included in these analyses obtained informed consent from individuals prior to participation. The primary aim was to investigate whether HbA1c was affected by the intensity/volume of aerobic exercise training and therefore we only included studies that measured HbA1c. Where an investigation took multiple measures (fasting blood glucose, total cholesterol, triglycerides, high-density lipoprotein [HDL], and low-density lipoprotein [LDL]), we included them as separate data sets. Full-text articles and supplemental data were assessed for methodological quality using the PEDro (Physiotherapy Evidence Database) scale [26]. To determine whether there was any publication bias, funnel plots for each outcome variable were constructed [27].

### Outcomes

The data from each article were extracted for the change in variables pre- and post-exercise intervention including: HbA1c, fasting blood glucose, total cholesterol, HDL cholesterol, LDL cholesterol, triglycerides, and BMI. Changes in HbA1c, the gold-standard measure of glycaemic control [28], rely on the turnover of red blood cells, which glucose binds to irreversibly [6]. The half-life of red blood cells is 12 weeks so only interventions of ≥ 12 weeks were included which measure HbA1c [29]. Aerobic exercise interventions included walking (*n* = 5), running (*n* = 6), and other (interval training, cycling) (*n* = 5), in intensities ranging from 30 to 80% of maximal exercise capacity. The intensity of the exercise stimulus progressed throughout some interventions and thus, the average intensity across the duration of each programme ranges from 50 to 75%. Details of the studies included are described in Table 1. Missing change in standard deviation values was predicted using the following formula (where the correlation coefficient (*Corr*) was calculated from studies where the change in standard deviation was included) [30]:

$${\text{Corr}} = \frac{{{\text{SD}}^{2}_{{{\text{baseline}}}} + {\text{SD}}^{2}_{{{\text{final}}}} - {\text{SD}}_{{{\text{change}}}} }}{{\left( {2 \times {\text{SD}}_{{{\text{baseline}}}} \times {\text{SD}}_{{{\text{final}}}} } \right)}}$$

$${\text{SD}}_{{{\text{change}}}} = \sqrt {{\text{SD}}^{2}_{{{\text{baseline}}}} + {\text{SD}}^{2}_{{{\text{final}}}} - \left( {2 \times {\text{Corr}} \times {\text{SD}}_{{{\text{baseline}}}} \times {\text{SD}}_{{{\text{final}}}} } \right)}$$

**Table 1:** Description of included studies and data sets

Reference	Exercise Intervention	Design method	Outcome measures	Participants	PEDro score
[46]	Aerobic exercise-150 min total per week at 65% of maximal exercise capacity	RCT	HbA1c	Type 2 diabetics, control = 41, EX = 72	7/10
[66]	Aerobic exercise for 105 min total per week at an average of 58% total exercise capacity Three phases- phase 1 = 90 min at 50% V̇O_2_ max, phase 2 = 90 min at 60% V̇O_2_ max, phase 3 = 120 min at 65% V̇O_2_ max	RCT	HbA1c, fasting glucose, total cholesterol, triglycerides, LDL, HDL, BMI	Control = 15, EX = 14	6/10
[67]	Aerobic exercise averaged at 120 min per week, each session increased from 20 to 60 min throughout protocol Averaged at 65% V̇O_2_ max, started at 60% V̇O_2_ max and gradually increased to 70%	RCT	HbA1c, BMI, total cholesterol, fasting blood glucose	Control = 20, EX = 20	6/10
[68]	90 min per week at 65% V̇O_2_ max. Walking at ventilatory threshold	RCT	HbA1c	Control = 17, EX = 14	6/10
[38]	20–60 min sessions, increased in 10 min each week until 60 min in 5th week. Averaged at 120 min per week 30–40% max HR for two weeks then increased every 4 weeks. Averaged at 62.5%	RCT	HbA1c, total cholesterol, triglyceride, LDL, HDL, BMI	Control = 10, EX = 9	7/10
[69]	Each session 50 min, 2 × per week. 100 min per week at 53% exercise capacity based on HR corresponding to aerobic threshold	RCT	HbA1c, total cholesterol, LDL, HDL, BMI	Control = 15, EX = 15	5/10
[39]	Progressed from 15 min per session to 45 min per session, averaged at 100 min per week 60% max HR progressed to 75% max HR, averaging at 65% maximal exercise capacity	RCT	HbA1c, total cholesterol, triglyceride, LDL, HDL, BMI	Control = 63, EX = 60	7/10
[48]	Interval training averaging at 97.5 min per week and 58% maximal exercise capacity as jogging at 90–100% HR_reserve_ and walking < 70%	RCT	HbA1c, fasting glucose, total cholesterol, triglycerides, LDL, HDL, BMI	Control = 10, EX = 13	6/10
[42]	120 min exercise per week at 3.6–5.2mets = ~ 50% maximal exercise capacity	RCT	HbA1c, BMI	Control = 16, EX = 15	6/10
[43]	120 min per week, 75% maximal exercise capacity	RCT	HbA1c, total cholesterol, triglyceride, LDL, HDL, BMI	Control = 20, EX = 20	7/10
[51]	195 min per week at 65% maximal exercise capacity	RCT	HbA1c, fasting glucose, BMI	Control = 30, EX = 35	4/10
[52]	135 min per week at 60% maximal exercise capacity	RCT	HbA1c, fasting blood glucose, total cholesterol, LDL, HDL, BMI	Control = 11, EX = 12	7/10
[44]	180 min per week, 75% maximal exercise capacity (lactate threshold)	RCT	HbA1c, fasting blood glucose, total cholesterol, triglyceride, HDL, BMI	Control = 12, EX = 12	4/10
[41]	180 min per week at 56% maximal exercise capacity	RCT	HbA1c, fasting blood glucose, total cholesterol, triglycerides, LDL, HDL, BMI	Control = 9, EX = 10	5/10
[70]	90 min per week at 65% maximal exercise capacity	RCT	HbA1c, fasting blood glucose, total cholesterol, triglycerides, LDL, HDL, BMI	Control = 30, EX = 22	7/10
[71]	135 min per week averaging at 62.5% maximal exercise capacity	RCT	HbA1c, V̇O_2_ peak, BMI	Control = 10, EX = 31	6/10

HRmax, maximum heart rate; RCT, randomized control trial; HbA1c, Glycated Haemoglobin; BMI, body mass index; LDL, low-density lipoprotein; HDL, high-density lipoprotein

Means and standard deviation (SD) values not reported in text but present in figures within articles were determined using computer software (Image J, Maryland, USA, Imagej.net) [31]. Data were imported into a spreadsheet, which was specifically designed for meta-analyses (The jamovi project (2021), *jamovi* (Version 1.6) [Computer Software] retrieved from https://www.jamovi.org). Figures were prepared in jamovi and GIMP (GIMP 2.8.4, retrieved from https://www.gimp.org).

### Data quality assessment and statistical analysis

Heterogeneity, any variability within the studies, was quantified with the *I*^2^ statistic [32]. An *I*^2^ value of 25% may be interpreted as low, 50% as moderate and 75% as high between-study heterogeneity. A maximum likelihood (estimator: Tau^2^) random-effect meta-analysis approach was carried out on the aerobic exercise protocols, using the below equation:

$$ES_{i} = \theta_{i} + \in_{i} = \mu_{\theta } + \tau_{i} + \in_{i}$$

where ${\theta }_{i}$ = population effect size, ${\epsilon }_{i}$ = an error term by which ${ES}_{i}$ differs from ${\theta }_{i}$, ${\mu }_{\theta }$ = describes the central tendency, ${\tau }_{i}$ = an error term which the population effect size of the $i$ th study differs from ${\theta }_{i}$. [33]

Data extracted from each study included: study sample size, intervention/control group descriptions, study design, analysis method, and outcome data. Outliers were excluded if they had a studentised residual larger than the 100 × (1–0.05/(2 K))th percentile of a standard normal distribution where K is the number of studies [34]. Methodological quality was assessed using the modified 0–10 PEDro scale [35]. The primary outcome variables were defined as HbA1c pre- and post-intervention. Standardised mean differences (SMD) were calculated. Funnel plots were performed to assess publication bias with standard error plot against the standardised mean difference.

Random effects meta-regressions (continuous covariates) were conducted to explore the association of volume (time per week and total hours per intervention) and intensity (% maximal exercise capacity) of aerobic exercise with changes in HbA1c concentrations. Meta-regression analyses were carried out in R (The R foundation (2021), *R (version 4.1.0)* [computer software] retrieved from https://cran.r-project.org), using the SMD estimates of HbA1c and the two exercise programme variables- intensity and volume. The equation below was used to model the meta-regression:

$$\theta_{k}^{ \wedge } = \theta + \beta x_{k} + \in_{k} + \zeta_{k}$$

where *θ* = the intercept, *β* = regression weight, $x$ = continuous variable, $\in_{k}$ = sampling error, $\zeta_{k}$ = between-study heterogeneity [36].

Scatter bubble plots were constructed graphically to display proportional weights of different trials.

## Results

### Study selection

**Fig. 1:**
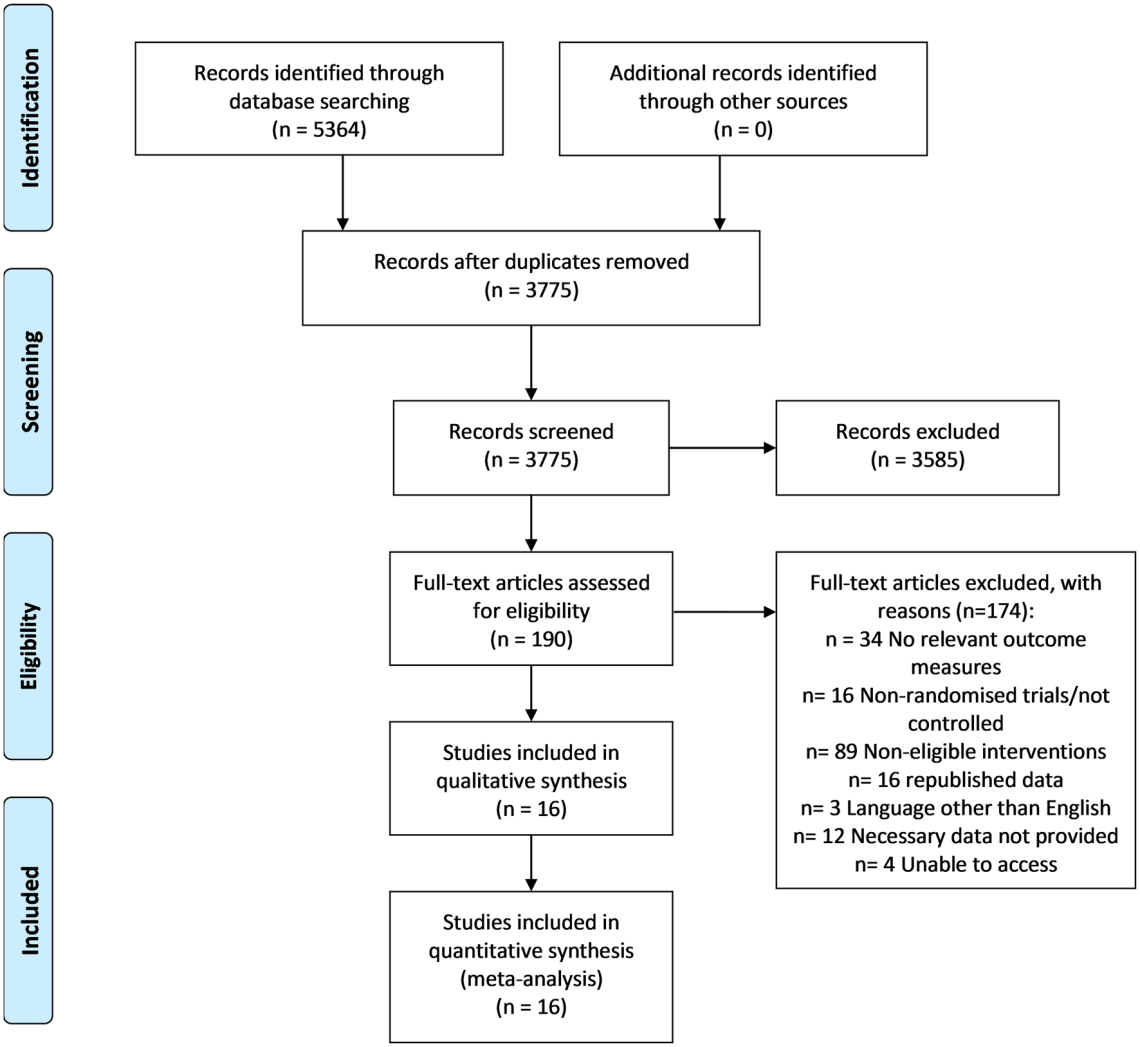
PRISMA flow diagram of studies included in the systematic review

After the initial database search, 5364 records were identified (Fig. 1). Once duplicates were removed, 3775 titles and abstracts were screened for inclusion resulting in 190 studies being retrieved as full text and assessed for eligibility. Of those, 170 were excluded and 20 articles remained, and due to missing details 16 studies were used in the final quantitative synthesis. To assess publication bias, funnel plots for aerobic exercise were computed and the trim and fill method was utilised to determine the number of studies required to balance the funnel plot [37].

### Description of the included trials

#### Trial setting and participants

The aerobic exercise programme settings included university health or research departments, community-based exercise facilities, and large public hospitals. The number of participants in each clinical trial ranged from *n* = 19 to *n* = 251 [38, 39]. One trial included only female participants [38] and one trial included only male participants [40]. All trials were carried out on adults and the age of participants ranged from 30 to 75 years. Baseline HbA1c (%) ranged from an average of 6.7% (50 mmol/mol) [41] to 8.8% (73 mmol/mol) [40] for all trials included. The average intensity of the exercise programmes (as % total exercise capacity) ranged from 50 [42] to 75% [43, 44]. The programme volume varied between 20 and 144 h in total and between 90 and 195 min per week. The interventions ranged from 50 min 2 × per week for 12 weeks [45] to 60 min 2 × per week for 72 weeks [43].

#### Interventions

**Fig. 2:**
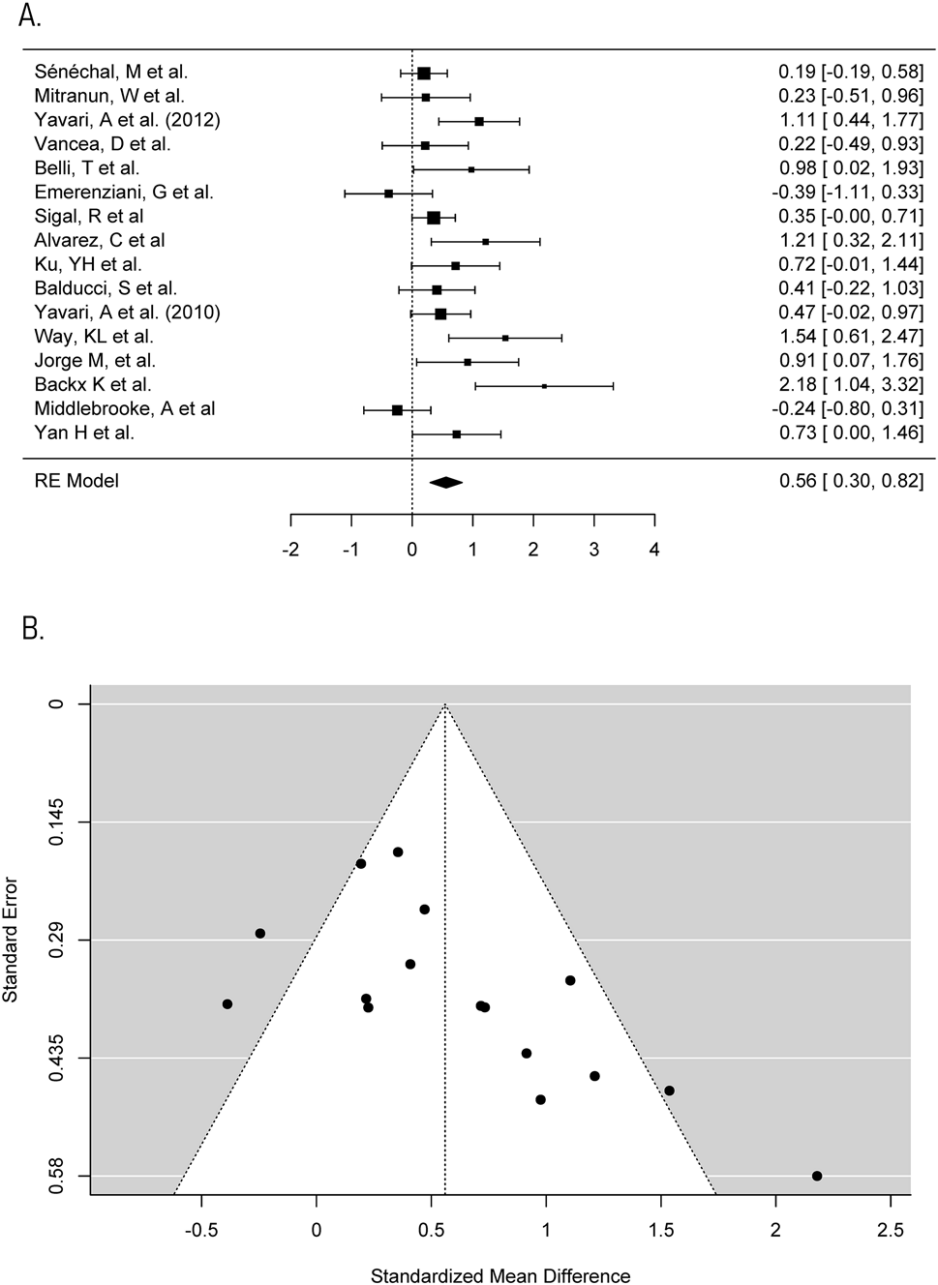
Meta-analysis of HbA1c **a** Forest plot comparing the effects of exercise on HbA1c concentrations (SMD). **b** Funnel plot of studies evaluating the effect of exercise on HbA1c with standardised mean difference plot against standard error which accounts for the sample size in the studies

**Fig. 3:**
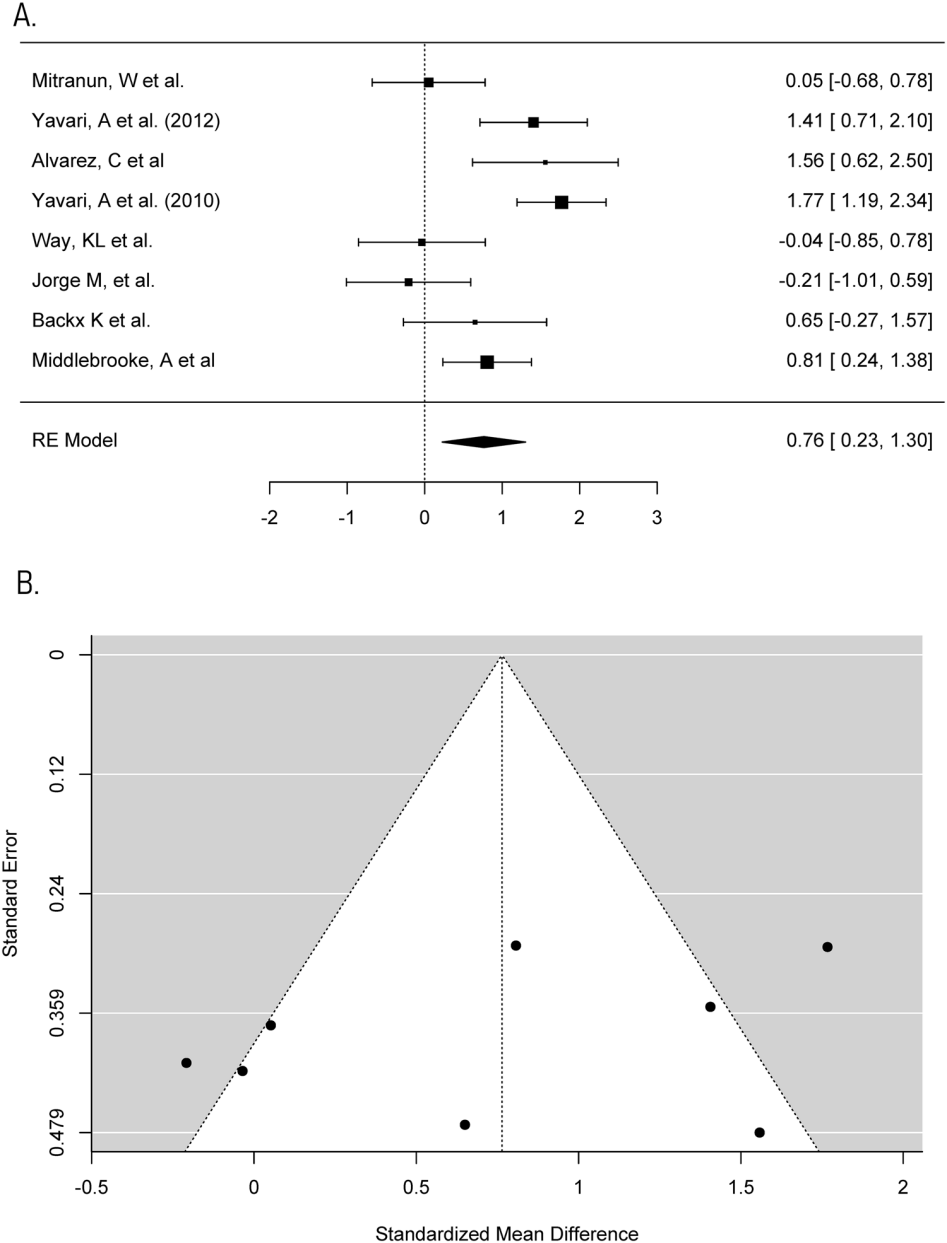
Meta-analysis of fasting blood glucose **a** Forest plot comparing the effects of exercise on fasting blood glucose concentrations (SMD). **b** Funnel plot of studies evaluating the effect of exercise on fasting blood glucose with standardised mean difference plot against standard error which accounts for the sample size in the studies

**Fig. 4:**
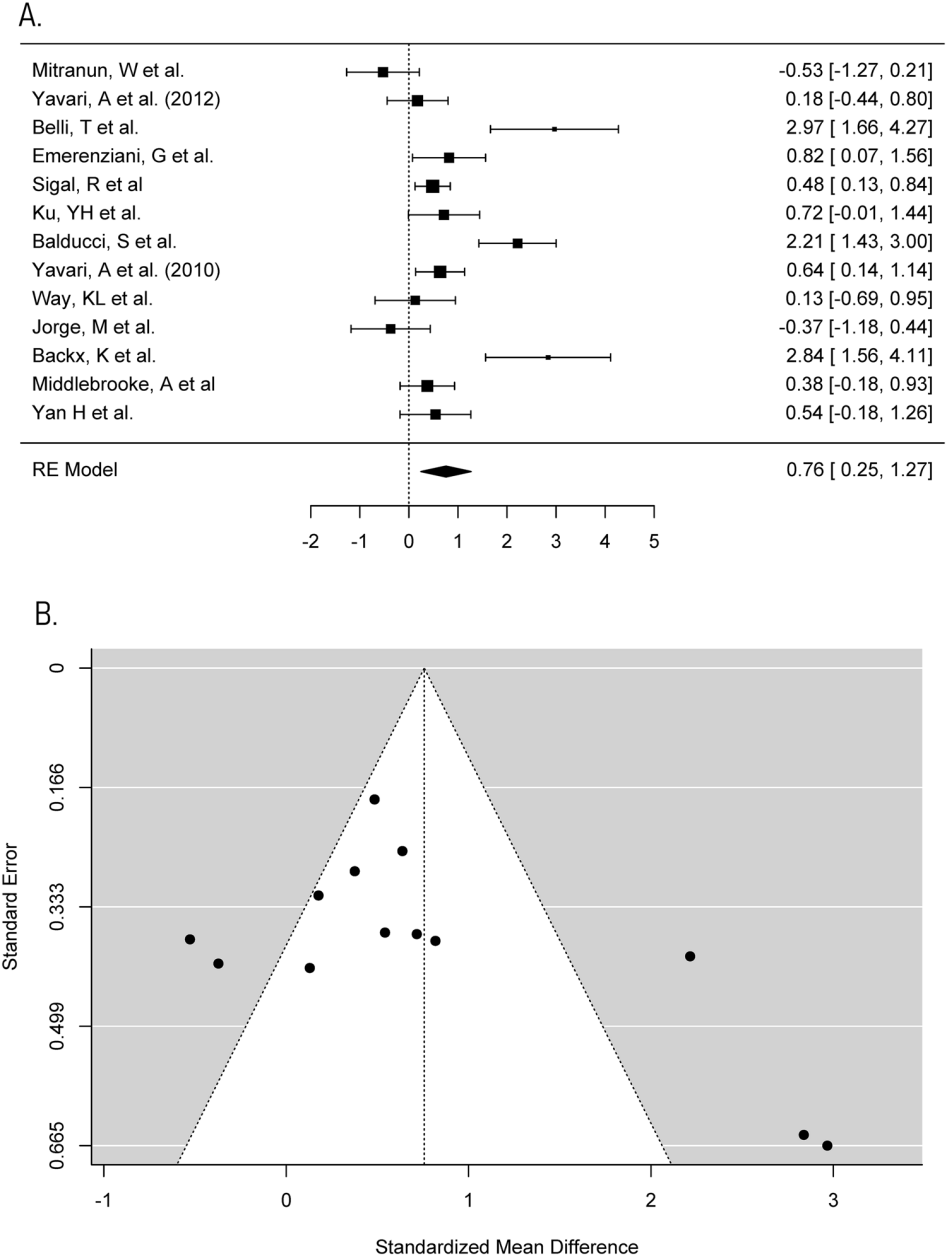
Meta-analysis of BMI **a** Forest plot comparing the effects of exercise on BMI (SMD). **b** Funnel plot of studies evaluating the effect of exercise on BMI with standardised mean difference plot against standard error which accounts for the sample size in the studies

**Fig. 5:**
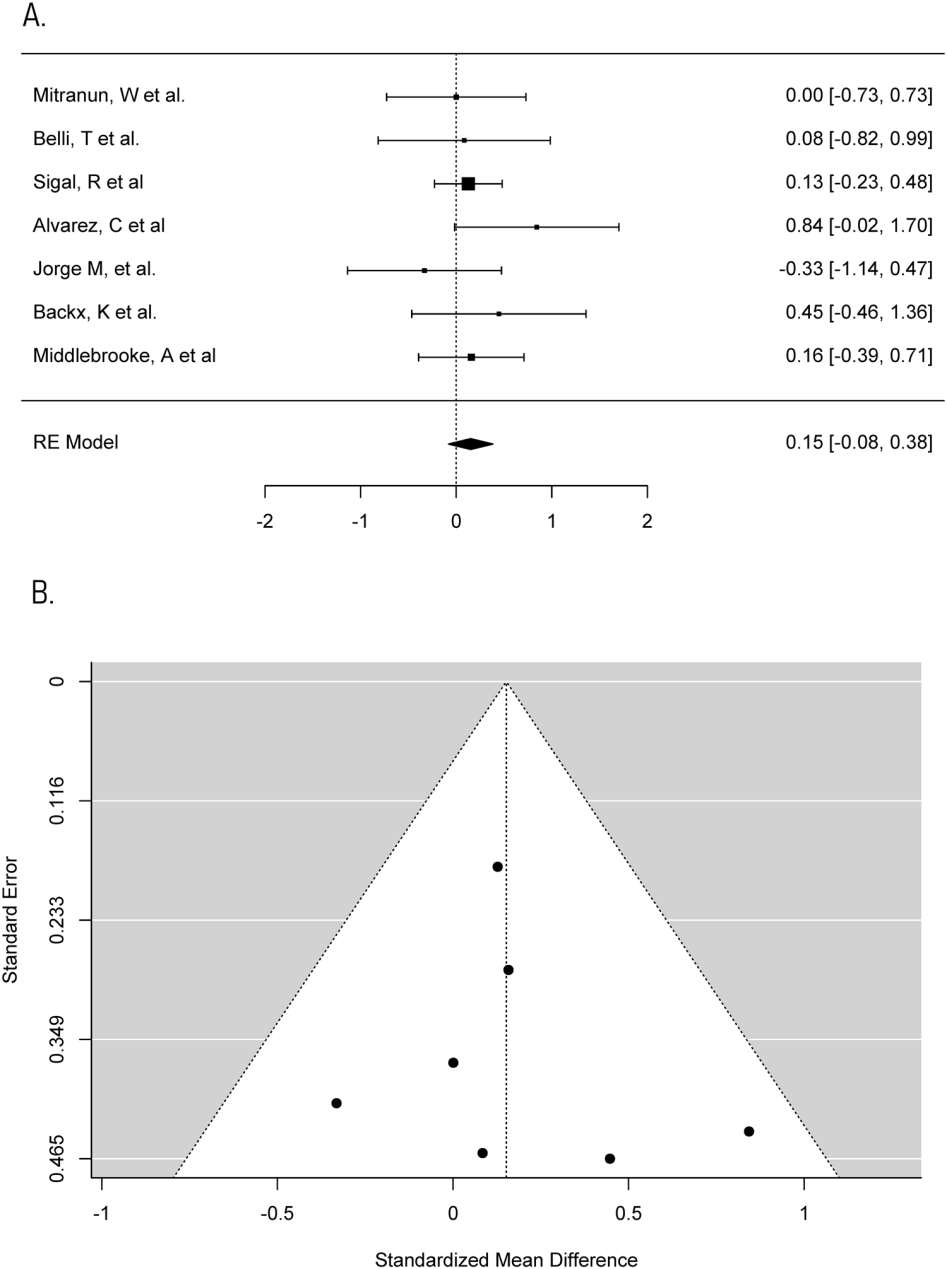
Meta-analysis of triglyceride concentrations **a** Forest plot comparing the effects of exercise on triglyceride concentrations (SMD). **b** Funnel plot of studies evaluating the effect of exercise on triglyceride concentrations with standardised mean difference plot against standard error which accounts for the sample size in the studies

A description of the exercise programmes included is given in Table 1. The duration of interventions ranged from 12 to 72 weeks, with a median of 12 weeks. The studies mainly included aerobic exercise in the form of cycling, jogging, and walking. Seventy-five per cent of studies included had three exercise sessions per week, two interventions had only two sessions per week [43, 45] and two interventions had up to five sessions per week [42, 46]. Each exercise session lasted between 20 min and 1 h, with most sessions lasting between 30 min and 1 h. All interventions were supervised (Figs. 2, 3, 4, 5).

### Synthesis of results

Of the 16 studies included, all were RCTs. Where a study had multiple conditions, they were treated separately.

#### Glycaemic control

HbA1c reflects a cumulative history of an individual’s blood glucose levels during the previous 2–3 months [6]. This meta-analysis investigated the effect of regular supervised aerobic exercise on change in HbA1c, as the gold-standard marker of glycaemic control [28]. All 16 trials provided data on HbA1c. The individual mean difference of pre–post-changes in HbA1c in control and aerobic groups, respectively, ranged from − 1.2 to 1.1% (median = − 0.09%) and − 1.3 to 0.1% (median = − 0.51%). Standardised mean difference (SMD) of within-group change in HbA1c between control and aerobic groups in each of the trials ranged from − 0.39 to 2.18. A maximum likelihood random effects model estimates the difference of within-group change in HbA1c between control and aerobic groups to be 0.56 (95% CI 0.3–0.82, *p* < 0.001), favouring aerobic exercise over control for reducing HbA1c concentration. There was considerable heterogeneity in this meta-analysis (*I*^*2*^ = 61.05%, *p* < 0.001).

Fasting blood glucose concentrations help to examine the change in glucose concentration between the start and end of the exercise programme [47]. Although this may not be representative of the average long-term change in glycaemic control like HbA1c, it was used in this meta-analysis as a secondary line of evidence to support the changes indicated by HbA1c [47]. Eight trials recorded fasting blood glucose concentrations and one trial was determined and excluded, as previously described, due to being a large outlier in the data set [34, 48]. Therefore, seven trials were included in this meta-analysis. The individual SMD of pre–post-changes in fasting blood glucose in control and aerobic groups, respectively, ranged from − 1.35 to 0.6 mmol/L (median = 0.5 mmol/L) and − 1.53 mmol/L to 0.15 mmol/L (median = − 0.99 mmol/L). The SMD of within-group change in fasting blood glucose between control and aerobic groups in each of the trials ranged from − 0.21 to 1.77. A maximum likelihood random effects model estimates the difference of within-group change in fasting blood glucose between control and aerobic groups to be 0.76 (95% CI 0.23–1.3, *p* < 0.05). There was considerable heterogeneity in this meta-analysis (*I*^*2*^ = 76.67%, *p* < 0.001).

#### Anthropometric measures—BMI

Thirteen trials recorded the change in BMI after the exercise programme. The individual mean difference of pre–post-changes in BMI in control and aerobic groups, respectively, ranged from − 0.7 to 0.4 kg/m^2^ (median = − 0.1 kg/m^2^) and − 2.2 kg/m^2^ to 0.2 kg/m^2^ (median = − 0.8 kg/m^2^). The SMD of within-group change in BMI between control and aerobic groups in each of the trials ranged from − 0.53 to 2.97. A maximum likelihood random effects model estimates the difference of within-group change in BMI between control and aerobic groups to be 0.76 (95% CI 0.25–1.27, *p* < 0.05), and therefore the exercise programmes significantly reduced BMI. There was considerable heterogeneity in this meta-analysis (*I*^*2*^ = 86.35%, *p* < 0.001).

#### Lipid profiles—triglycerides

Increased concentrations of triglycerides result in lipotoxicity, exacerbate hyperglycaemia, and help to cause the diabetic dyslipidaemia associated with increased cardiovascular disease risk [49]. This meta-analysis therefore analysed the effect supervised aerobic exercise programmes have on triglyceride concentrations in those with type 2 diabetes to investigate how significantly exercise can be used as a treatment. Eight trials recorded triglyceride concentrations and one trial was calculated but ultimately excluded, as previously described, due to being a large outlier [34, 43]. This meta-analysis was therefore carried out on seven trials. The individual mean difference of pre–post-changes in triglyceride concentration in control and aerobic groups, respectively, ranged from − 0.58 to 0.20 mmol/L (median = − 0.05 mmol/L) and − 0.48 mmol/L to 0.00 mmol/L (median = − 0.16 mmol/L). SMD of within-group change in triglycerides between control and aerobic groups in each of the trials ranged from − 0.33 to 0.84. A maximum likelihood random effects model estimates the difference of within-group change in triglycerides between control and aerobic groups to be 0.15 (95% CI − 0.08 to 0.38) with *p* > 0.05. Thus, the exercise programmes did not significantly reduce triglyceride concentrations. There was no heterogeneity in the meta-analysis (*I*^*2*^ = 0%, *p* > 0.05).

#### Total cholesterol

**Fig. 6:**
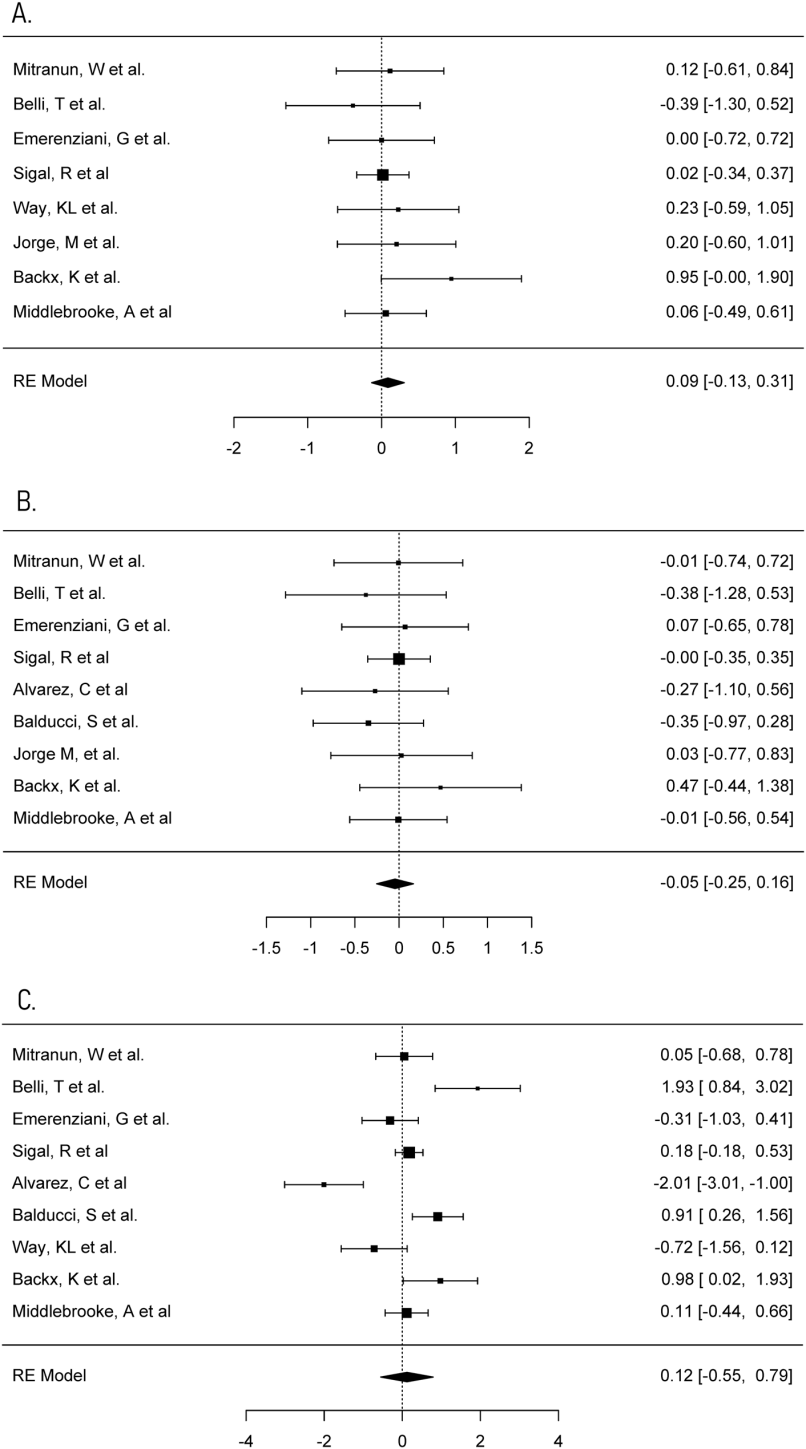
Meta-analysis of total cholesterol, HDL, and LDL concentrations. **a** Forest plot comparing the effects of exercise on total cholesterol concentrations (SMD). **b** Forest plot comparing the effects of exercise on HDL concentrations (SMD). **c** Forest plot comparing the effects of exercise on LDL concentrations (SMD)

Total cholesterol is a summation of the concentration of high-density lipoprotein (HDL) and low-density lipoprotein (LDL) [50]. It plays a role in increasing the risk of cardiovascular disease in those with type 2 diabetes [50]. This meta-analysis analysed whether the total cholesterol concentration changed after a period of supervised aerobic exercise as this may be a beneficial way to decrease the risk of cardiovascular disease in these individuals. Eleven trials reported total cholesterol concentration and three trials were determined and excluded, as previously described, due to being large outliers [34, 43, 48, 51]. This meta-analysis was therefore carried out on eight trials. The individual SMD of pre–post-changes in total cholesterol concentration in control and aerobic groups, respectively, ranged from − 0.35 to 0.33 mmol/L (median = 0.05 mmol/L) and − 0.70 mmol/L to 0.16 mmol/L (median = − 0.11 mmol/L). SMD of within-group change in total cholesterol between control and aerobic groups in each of the trials ranged from -0.39 to 0.95. A maximum likelihood random effects model estimates the difference of within-group change in total cholesterol between control and aerobic groups to be 0.09 (95% CI − 0.13 to 0.31, *p* > 0.05), and therefore the exercise programmes did not significantly reduce total cholesterol (Fig. 6a). There was no substantial heterogeneity in this meta-analysis (*I*^*2*^ = 0%, *p* > 0.05).

#### High-density lipoprotein

This meta-analysis investigated whether a period of supervised aerobic exercise had beneficial effects on increasing HDL and reducing the risk of cardiovascular disease. Ten trials recorded high-density lipoprotein (HDL) concentrations and one trial was determined and excluded, as previously described, due to being a large outlier [34, 52]. The individual SMD of pre–post-changes in HDL concentration in control and aerobic groups, respectively, ranged from − 0.06 to 0.20 mmol/L (median = 0.02 mmol/L) and − 0.15 mmol/L to 0.50 mmol/L (median = 0.07 mmol/L). The SMD of within-group change in HDL between control and aerobic groups in each of the trials ranged from -0.38 to 0.47. A maximum likelihood random effects model estimates the difference of within-group change in HDL between control and aerobic groups to be − 0.05 (95% CI − 0.25 to 0.16) with *p* > 0.05, and therefore the exercise programmes did not impact upon HDL (Fig. 6b). There was considerable heterogeneity in this meta-analysis (*I*^*2*^ = 83.08%, *p* < 0.001).

#### Low-density lipoprotein

We measured low-density lipoprotein (LDL) as it is significantly associated with the pathogenesis of type 2 diabetes. This meta-analysis investigated whether a programme of aerobic exercise reduces the increased concentrations of LDL in type 2 diabetes. Nine trials recorded low-density lipoprotein concentrations. The individual SMD of pre–post-changes in LDL concentration in control and aerobic groups, respectively, ranged from − 0.83 to 0.09 mmol/L (median = − 0.06 mmol/L) and − 1.3 mmol/L to 0.35 mmol/L (median = − 0.125 mmol/L). SMD of within-group change in LDL between control and aerobic groups in each of the trials ranged from − 2.01 to 1.93. A maximum likelihood random effects model estimates the difference of within-group change in LDL between control and aerobic groups to be 0.12 (95% CI − 0.55 to 0.79) with *p* > 0.05, and therefore the aerobic exercise programmes did not significantly change LDL (Fig. 6c). There was considerable heterogeneity in this meta-analysis (*I*^*2*^ = 88.31%, *p* < 0.001).

### Meta-regression

Meta-regression analyses were carried out to explore the association of volume and intensity of aerobic exercise with changes in HbA1c concentrations. All these analyses were weighted by the inverse of the variance of each observation and scatter bubble plots were constructed to graphically display proportional weights of different trials. Intensity (% maximal exercise capacity), volume (minutes per week), total volume (hours per intervention), and volume × intensity (minutes per week × % maximal exercise capacity) were all not significantly associated with changes in HbA1c (*p* = 0.96, 0.116, 0.853 and 0.602, respectively) and had moderate heterogeneity (*I*^*2*^ = 60.92%, 58.63%, 61.94% and 60.93%).

## Discussion

### Glycaemic control

**Fig. 7:**
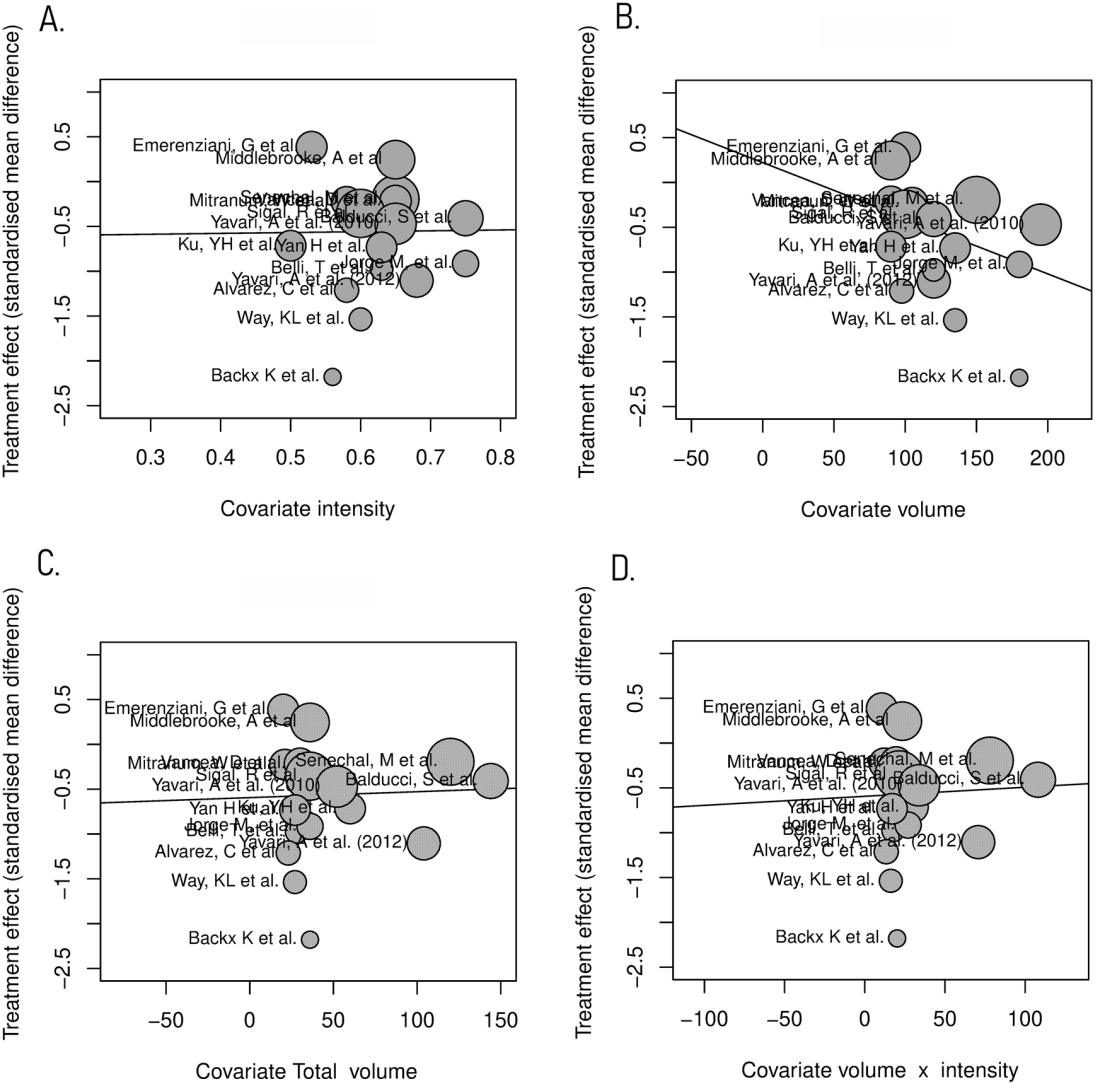
Meta-regression of **a** intensity, **b** volume (minutes per week), **c** volume (Total hours of intervention), and **d** volume (minutes per week) × intensity on HbA1c. Bubble plots showing the association of **a** intensity, **b** volume (minutes per week), **c** volume (Total hours of intervention), and **d** volume (minutes per week) × intensity on HbA1c concentrations after a programme of exercise

HbA1c significantly decreased in those with type 2 diabetes after ≥ 12 weeks supervised aerobic exercise. This meta-analysis shows that 87.5% of exercise interventions reduced HbA1c. The overall HbA1c SMD of the aerobic exercise interventions was 0.56 (95% CI 0.3–0.82), which indicates that the exercise programme is around 0.5 times more effective in decreasing HbA1c concentrations than the control group [53]. Aerobic exercise programmes of ≥ 12 weeks help regulate glycaemic control and are therefore likely to be beneficial in treating the disease and helping to reduce the risk of comorbidities. The improvement in HbA1c could be mediated through two pathways. Aerobic exercise does not require PI3K to prompt GLUT-4 translocation and thus, insulin resistant tissue will still take up glucose during exercise [15]. This helps to lower the high blood glucose concentrations associated with the disease, and evidence for this is provided by the reductions in HbA1c seen in this meta-analysis. Adiposity is strongly linked to insulin resistance, and therefore weight loss induced by the aerobic exercise programme (indicated through a reduction in BMI) may also increase insulin sensitivity. This allows insulin to work more effectively in providing glycaemic control and could be an additional explanation for the reduction in HbA1c (Fig. 7).

Fasting blood glucose also significantly reduced after an aerobic exercise programme of ≥ 12 weeks. Indeed, 75% of studies included in the meta-analysis showed a decrease in fasting blood glucose. Fasting blood glucose reflects the momentary glycaemic state pre- and post-exercise programme and this demonstrates blood glucose concentrations reduced after the exercise programme [47]. However, it is an acute measurement parameter and is therefore only indicative of glucose concentrations at the exact point of measurement, rather than an average change throughout like HbA1c [47]. Measurement of HbA1c is recommended by the American Diabetes Association as the standard of care for monitoring diabetes due to its direct correlation with average plasma glucose concentrations [6]. The 75% of studies which showed a positive decrease in blood glucose concentrations help to support the evidence provided by HbA1c, that exercise programmes have a beneficial effect on helping to reduce blood glucose concentrations and improve glycaemic control in those with type 2 diabetes.

### HbA1c and BMI association

The BMI of participants significantly decreased by an average of 0.6 kg/m^2^ after the exercise programme of ≥ 12 weeks. People with type 2 diabetes typically have a higher BMI, including increased visceral adiposity [54]. The average BMI of participants at the beginning of the trials included in this meta-analysis was 30.1 kg/m^2^ and 80% of studies included showed a significant decrease in BMI post-exercise programme by 2.1% on average. The overall BMI SMD of the aerobic exercise interventions was 0.76 (95% CI 0.25–1.27), which suggests that the exercise programmes significantly decreased BMI by a magnitude of 0.76 times more than the no exercise control group. In those with type 2 diabetes, even small reductions (around 5%) in body mass have been proven to have significant impacts on improving glycaemic control [55]. For example, 0.1% reductions in HbA1c have been found with each 1 kg of body mass lost in patients with the disease [56]. It is difficult to determine whether exercise or weight loss has the biggest impact on glycaemic control as both factors are interrelated and as shown by this meta-analysis, exercise both reduces BMI and improves glycaemic control.

Improvements in BMI have also been associated with reducing the risk of mortality from comorbidities such as cardiovascular disease [55]. Weight loss has been associated with a 25% reduction in mortality risk in those with type 2 diabetes and a 28% reduction in the risk of mortality from cardiovascular disease after a 12-year prospective analysis study on 4970 overweight individuals with the disease was carried out [57]. This meta-analysis therefore provides further evidence that aerobic exercise programmes of ≥ 12 weeks, which have beneficial impacts on weight loss, may also reduce the risk of comorbidities associated with the disease. Weight loss by increased energy output has been shown to improve lipid profiles, including total cholesterol, HDL, LDL, and triglycerides, in overweight individuals at high risk of CVD, such as those with type 2 diabetes [58]. Evidence for significant improvements in cholesterol concentrations has been found when approximately 7% weight loss is maintained [58].

### Lipid profiles

Out of all the lipid profiles analysed, none changed significantly. This suggests that exercise programmes of this length don’t have a beneficial impact on triglyceride, total cholesterol, HDL and LDL concentrations. This could be due to the variation in programme length, and volume, frequency, and intensity of exercise prescribed to participants within the trials included within the meta-analysis. It could also have been impacted by participants taking certain medications. LDL concentrations are reduced by statins and evidence shows that LDL concentrations are no more improved with statins and exercise than with statins alone [59]. This could contribute to the non-significant results observed. Mitigating the impact of statin use on the analysis is challenging as excluding trials where any participants take statins would significantly reduce the sample size of trials included.

From the analysis, HDL and LDL show a large amount of heterogeneity (*I*^*2*^ = 83.08% and 88.31%, respectively), suggesting that the variation within trials, for example statin use, might impact the significance seen in these results [60]. Total cholesterol and triglyceride concentrations show low heterogeneity (*I*^*2*^ = 0%) which implies that all studies included had a very similar effect on these two outcomes. Evidence has found that total energy expenditure and intensity are key factors in determining the impact an exercise programme has on lipid profiles [61]. Longer programmes at high intensity have proven to reduce total cholesterol and increase HDL cholesterol concentrations [62] and evidence for this is seen within some of the trials included within this meta-analysis. For example, Yavari et al. [51] carried out a protocol for 52 weeks, with 3 sessions per week at an average intensity of 67.5%. The SMD for this trial was 1.72 providing evidence that this longer and higher intensity programme reduced total cholesterol concentrations when compared with the control group. Moderate intensity exercise but at a higher volume (time per week) has also proven to reduce triglyceride concentrations and increase HDL concentrations as lipids account for the majority of oxidative metabolism at sustained low–moderate intensity [63]. Evidence suggests the most significant improvements are seen in high-volume and high-intensity programmes [64].

There is also some evidence that weight loss of approximately 7% has to be sustained before cholesterol concentrations significantly change [58]. The meta-analysis on BMI (Fig. 4) shows that the average reduction in BMI of participants carrying out the exercise interventions is 2.1%. As exercise and weight loss are interrelated, the weight loss, as a result of these interventions, might be insufficient for beneficial changes to occur in lipid concentrations. This suggests that some of the aerobic exercise interventions included might lack the intensity, volume, and weight loss required to provide beneficial changes in lipid profiles and therefore contribute to the non-significant results.

### Association of HbA1c with intensity and volume

The intensity of the aerobic exercise programmes included in this meta-analysis varied from an average of 50–75% of maximal exercise capacity. The programme volume varied between 20 and 144 h in total and between 90 and 195 min per week. The interventions ranged from 50 min 2 × per week for 12 weeks [45] to 60 min 2 × per week for 72 weeks [43]. A number of measures included in the meta-analyses demonstrate substantial statistical heterogeneity and this is likely due to differences within the protocols of each trial, including differences in intensity, volume, and type of exercise.

A meta-regression was carried out on HbA1c using intensity and volume as moderators to investigate the heterogeneity found and the effects these variables could have on glycaemic control. When used as separate moderators, intensity, volume (mins per week) and total volume (total hours of intervention) all had non-significant associations with HbA1c concentrations (*p* = 0.96, 0.116, 0.853, respectively). However, all three variables also have moderate heterogeneity within the results (*I*^*2*^ = 60.92%, 58.63%, and 61.94%). This could be due to variations within the studies, including medication use and differences in aerobic exercise programmes. Confidence in the significance could be improved by having a bigger sample size available to use within the meta-regression.

For the effects of aerobic exercise programmes to be better investigated within the meta-regression, trials need to investigate a bigger range of intensities. The trials included only range in intensity from 50 to 75% with a big proportion of exercise programmes ranging between intensities of 60–70%, this is therefore unlikely to give the true association of intensity with HbA1c. A meta-analysis investigating the difference between high-intensity interval training (HIIT) and moderate intensity interval training (MIIT) found that HIIT at 85–95% max HR showed a 0.37% greater reduction in HbA1c than MIIT [65]. Further supporting the idea that potentially, the range of exercise intensity used in this meta-regression was not great enough to show an association. The median length of trials included was also 12 weeks and therefore future longer-term studies may strengthen the conclusions which can be made regarding optimal exercise volume. A meta-regression was carried out combining volume and intensity as a moderator to investigate the association with HbA1c. The results of this meta-regression were also not significant, likely due to the reasons stated above.

### Limitations

Whilst the literature assessment was comprehensive, it is possible that studies may have been missed from the analysis. There was significant heterogeneity in a number of measures included. Whilst random effects modelling was chosen to mitigate this effect, this remains a limitation. Whilst some interventions included have achieved statistical significance for a range of outcomes, the change in the outcome required to have a beneficial impact on patients and to be considered clinically relevant remains to be fully determined. This therefore provides a limitation in the analysis of the results.

The range of intensities used in the trials included in the meta-regression was limited to between 50 and 75%. Although this forms a limitation, it is likely that the interventions have not used lower intensities as the time per week required to exercise becomes unfeasibly long for type 2 diabetes patients to complete. It is also likely that the average intensity was limited to 75% as type 2 diabetes patients are sedentary individuals and therefore anything higher than 75% would be extremely challenging. The non-significant results from this meta-regression nonetheless provide interesting findings that longer and higher intensity exercise programmes may not confer additional benefits to glycaemic control in people with type 2 diabetes.

## Conclusion

Although there is substantial evidence indicating the benefits of aerobic exercise as a treatment for those with type 2 diabetes, there is still insufficient evidence on the exact intensity, volume, and duration of exercise required to provide optimal glycaemic control. This systematic review and meta-analysis provide evidence that short-term interventions begin to show improvements in glycaemic control, but long-term interventions might be required to provide beneficial changes in lipid profiles and therefore further research needs to be carried out into longer-term interventions. This meta-regression shows that higher volume or more intense exercise interventions might not confer additional benefits to glycaemic control in people with type 2 diabetes. However, further research needs to be carried out on a wider range of interventions to reach more confident conclusions.

## Supplementary Information

Below is the link to the electronic supplementary material.

Supplementary file1 (DOCX 13 kb)
