# Supplementary material for: There is no dose–response relationship between the amount of exercise and improvement in HbA1c in interventions over 12 weeks in patients with type 2 diabetes: a meta-analysis and meta-regression
Source: Acta Diabetol. 2022 Aug 5;59(11):1399–415. doi: 10.1007/s00592-022-01918-8 (PMC9519659; doi:10.1007/s00592-022-01918-8)
Supplement: Supplementary file 1 — Supplementary file1 (DOCX 13 kb) [file 592_2022_1918_MOESM1_ESM.docx]

**Supplementary material. 1** Detailed PubMed search terms

("glycemic control"[MeSH Terms] OR ("glycemic"[All Fields] AND "control"[All Fields]) OR "glycemic control"[All Fields] OR ("glycaemic"[All Fields] AND "control"[All Fields]) OR "glycaemic control"[All Fields]) AND ("diabetes mellitus, type 2"[MeSH Terms] OR "type 2 diabetes mellitus"[All Fields] OR "type 2 diabetes"[All Fields]) AND ("exercise"[MeSH Terms] OR "exercise"[All Fields] OR "exercises"[All Fields] OR "exercise therapy"[MeSH Terms] OR ("exercise"[All Fields] AND "therapy"[All Fields]) OR "exercise therapy"[All Fields] OR "exercise s"[All Fields] OR "exercised"[All Fields] OR "exerciser"[All Fields] OR "exercisers"[All Fields] OR "exercising"[All Fields]) AND (("random allocation"[MeSH Terms] OR ("random"[All Fields] AND "allocation"[All Fields]) OR "random allocation"[All Fields] OR "random"[All Fields] OR "randomization"[All Fields] OR "randomized"[All Fields] OR "randomisation"[All Fields] OR "randomisations"[All Fields] OR "randomise"[All Fields] OR "randomised"[All Fields] OR "randomising"[All Fields] OR "randomizations"[All Fields] OR "randomize"[All Fields] OR "randomizes"[All Fields] OR "randomizing"[All Fields] OR "randomness"[All Fields] OR "randoms"[All Fields]) AND ("controling"[All Fields] OR "controllability"[All Fields] OR "controllable"[All Fields] OR "controllably"[All Fields] OR "controller"[All Fields] OR "controller s"[All Fields] OR "controllers"[All Fields] OR "controlling"[All Fields] OR "controls"[All Fields] OR "prevention and control"[MeSH Subheading] OR ("prevention"[All Fields] AND "control"[All Fields]) OR "prevention and control"[All Fields] OR "control"[All Fields] OR "control groups"[MeSH Terms] OR ("control"[All Fields] AND "groups"[All Fields]) OR "control groups"[All Fields]) AND ("clinical trials as topic"[MeSH Terms] OR ("clinical"[All Fields] AND "trials"[All Fields] AND "topic"[All Fields]) OR "clinical trials as topic"[All Fields] OR "trial"[All Fields] OR "trial s"[All Fields] OR "trialed"[All Fields] OR "trialing"[All Fields] OR "trials"[All Fields]))

**Translations**

**glycaemic control:** "glycemic control"[MeSH Terms] OR ("glycemic"[All Fields] AND "control"[All Fields]) OR "glycemic control"[All Fields] OR ("glycaemic"[All Fields] AND "control"[All Fields]) OR "glycaemic control"[All Fields]

**type 2 diabetes:** "diabetes mellitus, type 2"[MeSH Terms] OR "type 2 diabetes mellitus"[All Fields] OR "type 2 diabetes"[All Fields]

**exercise:** "exercise"[MeSH Terms] OR "exercise"[All Fields] OR "exercises"[All Fields] OR "exercise therapy"[MeSH Terms] OR ("exercise"[All Fields] AND "therapy"[All Fields]) OR "exercise therapy"[All Fields] OR "exercise's"[All Fields] OR "exercised"[All Fields] OR "exerciser"[All Fields] OR "exercisers"[All Fields] OR "exercising"[All Fields]

**randomised:** "random allocation"[MeSH Terms] OR ("random"[All Fields] AND "allocation"[All Fields]) OR "random allocation"[All Fields] OR "random"[All Fields] OR "randomization"[All Fields] OR "randomized"[All Fields] OR "randomisation"[All Fields] OR "randomisations"[All Fields] OR "randomise"[All Fields] OR "randomised"[All Fields] OR "randomising"[All Fields] OR "randomizations"[All Fields] OR "randomize"[All Fields] OR "randomizes"[All Fields] OR "randomizing"[All Fields] OR "randomness"[All Fields] OR "randoms"[All Fields]

**control:** "controling"[All Fields] OR "controllability"[All Fields] OR "controllable"[All Fields] OR "controllably"[All Fields] OR "controller"[All Fields] OR "controller's"[All Fields] OR "controllers"[All Fields] OR "controlling"[All Fields] OR "controls"[All Fields] OR "prevention and control"[Subheading] OR ("prevention"[All Fields] AND "control"[All Fields]) OR "prevention and control"[All Fields] OR "control"[All Fields] OR "control groups"[MeSH Terms] OR ("control"[All Fields] AND "groups"[All Fields]) OR "control groups"[All Fields]

**trial:** "clinical trials as topic"[MeSH Terms] OR ("clinical"[All Fields] AND "trials"[All Fields] AND "topic"[All Fields]) OR "clinical trials as topic"[All Fields] OR "trial"[All Fields] OR "trial's"[All Fields] OR "trialed"[All Fields] OR "trialing"[All Fields] OR "trials"[All Fields]
